# The association between non-breast and ovary cancers and BRCA mutation in first- and second-degree relatives of high-risk breast cancer patients: a large-scale study of Koreans

**DOI:** 10.1186/s13053-018-0103-3

**Published:** 2019-01-03

**Authors:** Hakyoung Kim, Doo Ho Choi, Won Park, Young-Hyuck Im, Jin Seok Ahn, Yeon Hee Park, Seok Jin Nam, Seok Won Kim, Jeong Eon Lee, Jong Hwan Yu, Se Kyung Lee, Boo Yeon Jung

**Affiliations:** 1 Departments of Radiation Oncology, Samsung Medical Center, Sungkyunkwan University School of Medicine, 81 Irwon-Ro, Gangnam-gu, Seoul, 135-710 Republic of Korea; 2 Departments of Medicine, Samsung Medical Center, Sungkyunkwan University School of Medicine, Seoul, Republic of Korea; 3 Departments of Surgery, Samsung Medical Center, Sungkyunkwan University School of Medicine, Seoul, Republic of Korea

**Keywords:** Breast cancer, BRCA mutation, Family history, Genetic counseling

## Abstract

**Background:**

As a large-scale study of Koreans, we evaluated the association between BRCA mutation and the prevalence of non-breast and ovary cancers in first- and second-degree relatives of high-risk breast cancer patients.

**Methods:**

We organized familial pedigrees of 2555 patients with breast cancer who underwent genetic screening for *BRCA1/2* in Samsung Medical Center between January 2002 and May 2018. Families with a member that had a history of cancer other than of the breast or ovary were regarded positive for other primary cancer.

**Results:**

The median age of the population was 40 years (range, 19 to 82 years). BRCA mutation was detected in 377 (14.8%) of the patients. The BRCA-positive group had a higher frequency of family history of breast or ovarian cancer (*p* <  0.001), bilateral breast cancer (*p* = 0.021), and the male gender (*p* = 0.038). There were 103 (27.3%) patients who had multiple risk factors in the BRCA-positive group, while there were 165 (7.6%) patients who had multiple risk factors in the BRCA-negative group (*p* <  0.001). BRCA mutation was detected in 215 (11.7%) of the 1841 families without history of other primary cancers. Among the 714 families with histories of other primary cancers, 162 (22.7%) had BRCA mutation, and this was significantly more frequent (*p* < 0.001) than in those without a history. The occurrence of other primary cancers in families of high-risk patients was associated with a younger age at diagnosis (*p* = 0.044), bilateral breast cancer (*p* = 0.006), and BRCA mutations (p < 0.001). The most common site for the occurrence of another type of primary cancer was the stomach. In the BRCA-positive group, the proportional incidences of stomach, pancreas, colorectal, lung, and uterine cancer were 13.8, 4.0, 7.7, 8.8, and 5.0%, respectively; these were all relatively higher than those in the BRCA-negative group.

**Conclusions:**

We confirmed that BRCA mutation was associated with having multiple risk factors and an increased prevalence of non-breast and ovary cancers in first- and second-degree relatives of high-risk breast cancer patients. Due to the possibility of inherited cancer risk, genetic counseling with options for risk assessment and management should be provided to both patients and families of BRCA mutation carriers.

## Background

A BRCA mutation was defined as a mutation in either the *BRCA1* gene or *BRCA2* gene, which are tumor suppressor genes involved in various pathways, including cell-cycle propagation, DNA repair, and apoptosis. Pathogenic variant in these genes may provoke a hereditary breast-ovarian cancer syndrome in BRCA-mutation carriers. This mutation predisposed patients to other primary cancers such as stomach, pancreas, uterus, and prostate cancer [1–4]. Familial cancers other than breast and ovarian cancer were also associated with mutations of the *BRCA1* and *BRCA2* genes [5–8]. Taken together, BRCA mutations affect the development of secondary primary cancers other than that of the breast or ovary. One study showed a higher risk of secondary cancers among patients with a pathogenic variant [9]. However, previous studies have mainly focused on comparing patient and tumor characteristics, survivals, recurrence patterns, and risk of secondary cancer development according to BRCA mutation status [10–14]. Against this background, Noh et al. [15] showed that BRCA mutations in high-risk breast cancer patients were associated with having multiple family members with other primary cancers, despite the small number of eligible patients studied.

As one of the biggest general hospitals in South Korea, we cover nationally representative patient populations. In this current study, we actively performed genetic screening for *BRCA1/2* mutations in breast cancer patients who carried at least one of the risk factors, such as breast cancer with family history, bilateral breast cancer, breast cancer with family history of ovarian cancer, male breast cancer, or diagnosis before turning 40 years old. As a large-scale study of Koreans, the purpose of this study is to demonstrate the association between BRCA mutation and the prevalence of non-breast and ovary cancers in first- and second-degree relatives of high-risk breast cancer patients.

## Methods

### Patients

From January 2002 to May 2018, a total of 2555 patients were enrolled at a single institute, the Samsung Medical Center. Genetic screening was performed in patients who met the criteria of the National Health Insurance System of Korea, including breast cancer with family history, bilateral breast cancer, breast cancer with family history of ovarian cancer, male breast cancer, or diagnosis before turning 40 years old. The members of the family included first- and second-degree relatives, as well as the probands themselves. Following approval by the Institutional Review Board and the acquisition of informed consent, genetic counseling was primarily focused on personal and family history of any kind of cancers. The familial pedigree was organized by a research nurse. If any member had a history of cancer other than that of the breast or ovary, the family was regarded as positive for other primary cancers.

BRCA mutation analysis was conducted mainly at the Department of Laboratory Medicine and Genetics at Samsung Medical Center with the cooperation of three other DNA testing laboratories, all of which are certified annually by the Korean Institute of Genetic Testing Evaluation. Genomic DNA was extracted and purified from peripheral blood leukocytes. The whole exons and the flanking intrinsic sequences of the *BRCA1* gene or *BRCA2* gene were amplified by polymerase chain reaction. The amplified products were directly sequenced, and the sequences were then compared with reference sequences using Sequencher software (Gene Codes Co., Ann Arbor, USA). The nomenclature for BIC (Breast cancer Information Core) traditional mutations is used, based on U14680 (*BRCA1*) and U43746 (*BRCA2*). In addition, all mutations are described according to HUGO-approved systematic nomenclature (Nomenclature for the description of sequence variations, Human Genome Variation Society. http://www.hgvs.org/mutnomen/). HUGO-approved mutation nomenclature of *BRCA1* (GenBank accession no. NP_009225.1) and *BRCA2* (GenBank accession no. NP_000050.2) defines the A of the ATG translation initiation codon as nucleotide + 1. Splicing-defect mutations in intronic region are described at the genomic DNA level using GenBank genomic reference sequence NC_000017.10 (*BRCA1*) and NC_000013.10 (*BRCA2*). In addition, variants of unknown significance were excluded. Genetic testing of high-risk breast cancer patients was approved by the Institutional Review Board of Samsung Medical Center (2010–09–006-001).

### Statistical analysis

In order to analyze the relationships between the distributions of risk factors and BRCA mutations, chi-squared tests were used. These tests were also used to assess the relationships between the family history of other primary cancers. A *p*-value ≤0.05 was regarded as being indicative of statistical significance in two-tailed tests. In order to identify factors associated with family history of other primary cancers, a binary logistic regression analysis was performed. A Cochran-Mantel-Haenszel test was conducted in order to identify the distributions of frequently occurring familial cancers of patients according to BRCA mutation status. Statistical tests were performed using SPSS software, standard version 24.0 (IBM Corporation, Armonk, NY, USA).

## Results

The median age of the population was 40 years (range, 19 to 82 years). BRCA mutations were detected in 377 (14.8%) of the patients. Specifically, *BRCA1* gene or *BRCA2* gene mutations were detected in 186 (7.3%) and 196 (7.7%) patients, respectively. Among these, five patients had mutations in both *BRCA1* gene and *BRCA2* gene. Distributions of age and risk factors according to BRCA mutation status are described in Table 1. The BRCA-positive group had a higher frequency of family history of breast or ovarian cancer (*p* < 0.001), bilateral breast cancer (*p* = 0.021), and male gender (*p* = 0.038). There were 103 (27.3%) patients who had multiple risk factors in the BRCA-positive group, while there were 165 (7.6%) patients who had multiple risk factors in the BRCA-negative group (*p* < 0.001).

**Table 1:** Clinical characteristics according to BRCA mutation status (*n* = 2555)

Characteristics	BRCA (−) (*n* = 2178)	BRCA (+) (*n* = 377)	*p* value
Age [years; median (range)]	39 (19–82)	40 (23–75)	0.996
Risk factors			
Family history of breast or ovarian cancer			
No	1310 (60.1%)	105 (27.9%)	< 0.001
Yes	868 (39.9%)	272 (72.1%)	
Age at diagnosis			
> 40	1371 (62.9%)	245 (65.0%)	0.448
≤ 40	807 (37.1%)	132 (35.0%)	
Bilateral breast cancer			
No	1978 (90.8%)	328 (87.0%)	0.021
Yes	200 (9.2%)	49 (13.0%)	
Gender			
Female	2165 (99.4%)	371 (98.4%)	0.038
Male	13 (0.6%)	6 (1.6%)	
Number of risk factors			
Single	2013 (92.4%)	274 (72.7%)	< 0.001
Multiple	165 (7.6%)	103 (27.3%)	

The relationships between the distributions of risk factors and BRCA mutation were examined by chi-square test. The t-test was applied for comparing the distribution of age

There were 714 (28.0%) families that had a history of cancers other than breast or ovarian cancer. The distributions of families according to number of members having other primary cancers are described in Table 2. BRCA mutation was detected in 215 (11.7%) of the 1841 families without any history of other primary cancers. Among the 714 families with histories of other primary cancers, 162 (22.7%) had BRCA mutation; this was significantly more frequent than the proportion seen in families without such histories (*p* < 0.001). According to binary logistic regression, the occurrence of other primary cancers in families of high-risk patients was associated with a younger age at diagnosis (*p* = 0.044), bilateral breast cancer (*p* = 0.006), and BRCA mutations (*p* < 0.001), respectively (Table 3).

**Table 2:** Distributions of families according to number of members having other primary cancers (*n* = 2555)

Number of members having other primary cancers	BRCA (−) (*n* = 2178)	BRCA (+) (*n* = 377)	*p* value
0	1626 (74.7%)	215 (57.0%)	< 0.001
1	425 (19.5%)	114 (30.2%)	
2 or more	127 (5.8%)	48 (12.8%)

**Table 3:** Binary logistic regression analysis to identify factors associated with family history of other primary cancers

Response = Number of members having other primary cancers (0 vs. ≥1)	*p* value	Odds ratio (95% CI)
Variables		
Family history of breast or ovarian cancer	0.449	0.924 (0.753–1.134)
Younger age at diagnosis (≤40)	0.044	1.232 (1.006–1.508)
Bilateral breast cancer	0.006	1.517 (1.126–2.045)
Male gender	0.218	1.805 (0.706–4.618)
BRCA mutation	< 0.001	2.248 (1.773–2.850)

*CI* confidence interval

The most common site for the occurrence of another type of primary cancer was the stomach. In the BRCA-positive group, the proportional incidences of stomach, pancreas, colorectal, lung, and uterine cancer were 13.8, 4.0, 7.7, 8.8, and 5.0%, respectively; these were relatively higher than those of the BRCA-negative group (Table 4).

**Table 4:** Distributions of frequently occurring familial cancers of patients according to BRCA mutation status

Sites of other primary cancers	BRCA (−) (*n* = 2178)	BRCA (+) (*n* = 377)	Odds ratio (95% CI)	*p* value
Stomach	162 (7.4%)	52 (13.8%)	1.666 (1.183–2.345)	0.003
Pancreas	38 (1.7%)	15 (4.0%)	1.875 (1.006–3.494	0.048
Liver	107 (4.9%)	16 (4.2%)	0.656 (0.381–1.132)	0.130
Colorectal	96 (4.4%)	29 (7.7%)	1.661 (1.081–2.554)	0.021
Lung	109 (5.0%)	33 (8.8%)	1.586 (1.057–2.380)	0.026
Uterus	51 (2.3%)	19 (5.0%)	2.101 (1.216–3.631)	0.008

*CI* confidence interval

## Discussion

The carriers of *BRCA1* or *BRCA2* gene mutations have very high lifetime risks for breast and ovarian cancers. Since these genetic abnormalities can occur in other cancers, BRCA-associated pathways are critical in terms of safeguarding genetic content. Several previous studies in Korean breast cancer patients have been confined to either the prevalence of BRCA mutations or its association with risk factors and secondary malignancies [16–20]. With this background, the purpose of this study was to demonstrate the association between BRCA mutations and the prevalence of non-breast and ovary cancers in first- and second-degree relatives of high-risk breast cancer patients.

In terms of the risks of other primary cancers, Ford et al. [1] showed the risks of not only breast and ovarian cancer, but also other cancers in *BRCA1* gene mutation carriers. The estimated cumulative risk of breast cancer in gene carriers is 87% by age 70. Primary ovarian cancers occur in women with previous breast cancer, resulting in an estimated cumulative risk of ovarian cancer of 44% by age 70. Based on national incidence rates, significant excesses were observed for colon cancer (estimated relative risk (RR) to gene carriers 4.11 [95% confidence interval (CI) 2.36–7.15]) and prostate cancer (3.33 [1.78–6.20]); no significant excesses were noted for cancers of other sites. This study showed that carriers have increased risks for colon and prostate cancer, which provides clinical significance in certain family members if the risks are associated with particular mutations. Thompson et al. [3] conducted a cohort study to evaluate the risks of other cancers in *BRCA1* gene mutation carriers. *BRCA1* gene mutation carriers were found to have a statistically significant increase in risk for several cancers, including pancreatic cancer (RR = 2.26, 95% CI = 1.26 to 4.06, *p* = 0.004) and cancer of the uterine body and cervix (uterine body RR = 2.65, 95% CI = 1.69 to 4.16, *p* < 0.001; cervix RR = 3.72, 95% CI = 2.26 to 6.10, *p* < 0.001). There was some evidence of an elevated risk of prostate cancer in mutation carriers younger than 65 years old (RR = 1.82, 95% CI = 1.01 to 3.29, *p* = 0.05), but not in those 65 years old or older (RR = 0.84, 95% CI = 0.53 to 1.33, *p* = 0.45). Similarly, Easton et al. [21] showed the risks of certain cancers in *BRCA2* gene mutation carriers. Statistically significant increases in risks were observed for prostate cancer (estimated RR = 4.65; 95% CI = 3.48–6.22), pancreatic cancer (RR = 3.51; 95% CI = 1.87–6.58), gallbladder and bile duct cancer (RR = 4.97; 95% CI = 1.50–16.52), stomach cancer (RR = 2.59; 95% CI = 1.46–4.61), and malignant melanoma (RR = 2.58; 95% CI = 1.28–5.17). Friedenson et al. [4] evaluated the *BRCA1* gene and *BRCA2* gene pathways and the risk of cancers other than breast or ovarian. Although these mutations target the breast and ovary, a broader spectrum of cancers also occur with statistically significant elevated frequencies. Additional sites at risk include the stomach, pancreas, prostate, and colon. The increased risk ranged from about 20 to 60%, with the greatest risk increases occurring in the stomach and pancreas. In this background, Shih et al. [6] evaluated the incidence of BRCA mutations in breast cancer families with multiple primary cancers. Ninety-eight women selected from high-risk breast/ovarian cancer clinics with breast cancer reporting at least one other primary cancer in themselves or in a relative with breast cancer were compared to 99 women with breast cancer who reported a family history of breast cancer only. They concluded that the presence of multiple primary cancers predicted an increased likelihood of finding either a *BRCA1* or *BRCA2* gene mutation.

In this current study, we confirmed that BRCA mutation was detected more prevalently in families with histories of other primary cancers than those without any history of other primary cancers (22.7% vs. 11.7%, *p* < 0.001). Binary logistic regression analysis supported this finding that BRCA mutations was significantly associated with the occurrence of other primary cancers in families of high-risk patients (*p* < 0.001). A younger age at diagnosis (*p* = 0.044) and bilateral breast cancer (*p* = 0.006) were also associated with the occurrence of other primary cancers in families of high-risk patients. The most prevalent other primary cancer was stomach cancer, which was more common in South Korea than anywhere else in the world. In the BRCA-positive group, the proportional incidence of stomach cancer was 13.8%, which was relatively higher than that in the BRCA-negative group (7.4%, *p* = 0.003). Similar to previous studies, the risk of pancreatic cancer was higher among carriers of a BRCA mutation. BRCA mutations were also shown to be predisposing factors for the development of colorectal cancer, lung cancer, and uterine cancer in this study. Specifically, in the BRCA-positive group, the proportional incidences of pancreas, colorectal, lung, and uterine cancer were 4.0, 7.7, 8.8, and 5.0%, respectively; these were relatively higher than those in the BRCA-negative group. Not only environmental and genetic factors, but also cancer susceptibility from the BRCA mutation may contribute to the distribution of familial cancer. Our current study has a limitation: although the familial pedigree was organized thoroughly by a research nurse, the occurrence of other primary cancers in first- and second-degree relatives of high-risk patients could have been under-estimated due to a lack of information. Nonetheless, our current study supports previous studies suggesting that *BRCA1* gene and *BRCA2* gene mutations may be associated with increased susceptibilities to cancers other than breast and ovarian cancer. In addition to the large risks of breast and ovarian cancers, BRCA mutations may be associated with increased risks of several other cancers, particularly stomach, uterine, and pancreatic cancers.

## Conclusions

We confirmed that BRCA mutation was associated with having multiple risk factors in high-risk breast cancer patients. BRCA mutation was associated with increased prevalence of non-breast and ovary cancers in first- and second-degree relatives of high-risk breast cancer patients. Due to the possibility of inherited cancer risk, genetic counseling with options for risk assessment and management should be provided to both patients and families of BRCA mutation carriers.

## Abbreviations

CI: Confidence interval
RR: Relative risk
